# Climatic Variability Threatens Population Growth and Persistence of a Declining Grassland Songbird

**DOI:** 10.1002/ece3.72195

**Published:** 2025-11-24

**Authors:** N. E. Freeman, K. M. Silber, T. J. Hefley, A. M. Louthan, W. A. Boyle

**Affiliations:** ^1^ Division of Biology Kansas State University Manhattan Kansas USA; ^2^ School of Natural Sciences Bangor University Bangor Gwynedd UK; ^3^ Institute of Applied Ecology Sante Fe New Mexico USA; ^4^ Department of Statistics Kansas State University Manhattan Kansas USA; ^5^ Centre for Animals on the Move Western University London Ontario Canada

**Keywords:** fecundity, IPM, population projection, rainfall, sensitivity, survival

## Abstract

Determining the factors responsible for population change in threatened populations and the degree to which changing climates might put those populations at risk is one of the most pressing roles of conservation science. In the climatically variable grasslands of North America, songbirds are rapidly declining, and widespread habitat loss alone does not fully explain these declines. Theory predicts that increased variability in population growth rate, which could be generated by increased variability in weather conditions, should result in lower population sizes. We tested whether increasing variability in weather could have driven recent declines in songbird population numbers using detailed demographic data collected between 2013 and 2021 from grasshopper sparrows ( *Ammodramus savannarum* ) at the Konza Prairie Biological Station, Kansas, USA. To assess the effect of variability in weather on historical vital rates and population growth rate, we used results from an integrated population model to estimate sensitivity of population growth rate to weather and to conduct path analyses. We also used climate projections to predict population growth rate at our site and potential extirpation risk in a future climate. We found that historical population growth rate was over twice as sensitive to changes in adult apparent survival than other vital rates, and lagged population growth rate was lower following wetter years. Projections of future population size predicted that grasshopper sparrows may be locally extirpated within the next century, consistent with the expectation that increasingly variable precipitation patterns will reduce long‐term viability of songbird populations. Combining sophisticated modeling with detailed demographic data is critical for predicting trends in population growth and guiding conservation approaches in declining or at‐risk species.

## Graphical abstract

Determining the factors responsible for population change in threatened populations and the degree to which changing climates might put those populations at risk is one of the most pressing roles of conservation science. We tested whether increasing variability in weather could have driven recent declines in songbird population numbers using detailed demographic data collected between 2013 and 2021 from grasshopper sparrows (*Ammodramus savannarum*). Projections of future population size predicted that grasshopper sparrows may be locally extirpated in the next century, consistent with the expectation that increasingly variable precipitation patterns will reduce long‐term viability of songbird populations.

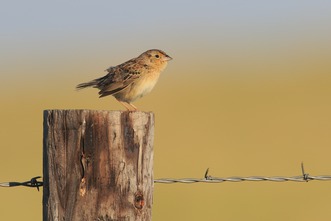

## 1 Background

Understanding the drivers of extinction or extirpation risk is a central problem in ecology and conservation biology. We know that temporal variation in population growth rate is a key determinant of long‐term population sizes, with high variability in annual population growth rate (*λ*) usually reducing stochastic growth rates (Boyce 1977; Tuljapurkar and Orzack 1980; Lande et al. 2003; Drake 2005). Assuming that annual population growth rate depends on environmental conditions (Andrewartha and Birch 1954; Drake and Lodge 2004; Jenouvrier et al. 2015), theory predicts that increasing variance in environmental conditions should decrease stochastic growth rates (Hilde et al. 2020). Such effects could occur in the absence of changes in mean environmental conditions. Because precipitation in many locations is predicted to increase in variability with little to no change in mean values (Easterling et al. 2017), it is particularly important to understand how variability in precipitation might influence population growth rate (Pendergrass et al. 2017). We lack empirical studies demonstrating how changes in precipitation variability might affect population dynamics (Lawson et al. 2015), limiting our ability to predict the consequences of changing climate regimes. In particular, we have a poor sense of how at‐risk species might respond to increased variability per se, especially in precipitation.

Increases in precipitation variability will only result in lower stochastic growth rates if the population growth rate depends on precipitation (and if this response is a linear or concave relationship; Lawson et al. 2015). However, populations occupying areas with highly variable precipitation regimes are likely to respond only weakly to precipitation due to the effects of demographic buffering (Pfister 1998), or to emigration from less favorable conditions. Because most studies of animal population dynamics are conducted in relatively stable environments (Caughley and Gunn 1993; Holmes 2007), we have little sense of whether animal populations that evolved in variable environments are likely to be highly responsive to that environmental variability. Effects of precipitation on the population growth rate could also be weak if effects are modulated through other trophic levels. For example, precipitation is the strongest driver of plant productivity in grasslands (Petrie et al. 2018) and affects plant, invertebrate, and vertebrate communities (Barnett and Facey 2016; Deguines et al. 2017; Dudney et al. 2017; Welti et al. 2020), meaning that precipitation effects on higher trophic levels might be mediated through precipitation effects on lower trophic levels. If lower trophic levels are strongly affected by other drivers besides precipitation, the linkage between precipitation and high trophic levels might be weak.

Complicating matters, the vital rates that comprise population growth rate could be affected by precipitation in potentially opposing or multiplicative ways. For example, local storms may reduce survival of endotherms because they experience elevated energetic costs of maintaining endothermy when wet (Newton 2007; Conrey et al. 2016; Wingfield et al. 2017). However, increased rainfall could positively impact fecundity or emigration via effects on vegetation structure or prey availability (Flanagan and Adkinson 2011; Silber et al. 2023), resulting in a net null effect of precipitation on population growth rate. Alternatively, if the relationship between precipitation and one or more vital rates is convex, increasing variability may actually increase stochastic population growth rate (Lawson et al. 2015). Thus, it is critical to identify the multiple ways in which precipitation might affect vital rates, and in turn, how vital rates affect population growth rate. Such effects are particularly important to understand if precipitation under climate change modifies some vital rates but not others. Continuing the storm example above, more storms in a future climate might result in lower survival, but if positive indirect effects of storms are not realized in a future climate due to temperature increases affecting vegetation structure or range shifts of prey, precipitation could reduce net population growth rate.

The grasslands of North America's central Great Plains are highly climatically variable (Knapp and Smith 2001) with high frequencies of multi‐decadal droughts. If populations respond to precipitation, precipitation will affect stochastic population growth rates (Maresh Nelson et al. 2024), and thus, extinction risk (Morris and Doak 2002). Here, we used 9 years of demographic data of a highly mobile bird species, grasshopper sparrows ( *Ammodramus savannarum* ) breeding in a tallgrass prairie of the central USA to quantify how population growth rate responds to annual variation in precipitation and understand how increases in precipitation variability will likely influence future population growth rates and risk of extirpation. First, we constructed an integrated population model (IPM) to estimate annual population growth, *λ*_t_, and annual vital rates (survival, fecundity, and immigration). Second, we constructed a simple stage‐structured population model using the vital rate estimates from the IPM to quantify the sensitivity of population growth rate to each vital rate. Third, we tested whether precipitation influenced vital rates and how those effects translated to population growth rate using a path analysis, including temperature in these models to account for differences in the consequences of rain under hot or cool conditions (Boyle et al. 2020). Finally, we forecast population counts at our site based on climate projections to estimate whether higher variability in precipitation under future climate regimes impacted stochastic population growth rate enough to affect extirpation risk.

## 2 Methods

### 2.1 Study Site and Species

We collected data on grasshopper sparrows at the Konza Prairie Biological Station (KPBS, 39°05′ N, 96°35′ W), a 3487‐ha tallgrass prairie in NE Kansas, USA. KPBS is composed of replicated pastures managed with different combinations of experimental grazing (grazed by bison or cattle, or ungrazed) and fire frequency (1, 2, 3, 4, or 20 year interval) treatments (Collins et al. 2021). In addition, we collected data at the Rannell's Preserve (RP), which is a 1175‐ha grassland immediately adjacent to KPBS with pastures that are grazed by cattle and burned annually (Owensby et al. 2008).

The grasshopper sparrow is a small, migratory songbird that breeds in grasslands throughout North America. They migrate annually, wintering in the southern United States and Mexico with the NE Kansas population wintering in Texas and northern Mexico (Hill and Renfrew 2019). In Texas, grasshopper sparrows have an overwinter survival probability ranging from 0.47 to 0.87 (Pérez‐Ordoñez et al. 2022). Over five decades, this species has declined at the rate of −2.48%/years., with even more rapid declines in recent years (“three generation” rate of −3.48%; North American Bird Conservation Initiative 2022). Range wide declines are mirrored in the regional data for the Great Plains in general and Eastern Kansas (Hostetler et al. 2023). Like many grassland‐obligate species, this species is faced with many environmental challenges, chief among them habitat loss due to agricultural conversion, woody plant encroachment, and management regimes that create homogeneous landscapes lacking critical vegetation features that support refugia from predators and severe weather (Bernath‐Plaisted et al. 2023). In the Flint Hills of Eastern Kansas, either over‐ or under burning are the chief sources of habitat degradation for grassland birds (Ratajczak et al. 2016; Winder et al. 2018).

On the breeding grounds, grasshopper sparrows occupy patchy grasslands with heterogeneous vegetation but few shrubs (Powell 2008; Vickery 2020). Grasshopper sparrows may attempt multiple nests each breeding season and can raise up to three broods per season at KPBS and RP (W.A.B., unpublished data). They construct domed ground nests of grasses and litter (Vickery 2020). Females lay and incubate 1–7 eggs and feed nestlings with the assistance of males until fledging at 7–8 days old (Vickery 2020; Winnicki 2019). Nest success (i.e., at least one sparrow fledging the nest) is low at the site (~23%), resulting in a mean of 0.18 fledglings per nest attempt (Verheijen et al. 2022), with failure primarily due to predation (snakes, mammals, birds; ~65% of field‐inferred causes of nest failures) and, to a lesser extent, abandonment, trampling, and flooding or other extreme weather events. At KPBS and RP, grasshopper sparrows were present from mid‐April through September, breeding occurred from May to July (occasionally into August), and both nests and adult sightings were most abundant in bison‐ and cattle‐grazed pastures that were burned every 2–3 years.

Grasshopper sparrows are generally territorial during the breeding season, but frequently disperse within and between breeding seasons. Within seasons, up to 75% of males change territories, with 52% of territories occupied by a new territorial male each month, and known within‐breeding‐season dispersal distances reached the maximum within‐site distance of 8.9 km (Williams and Boyle 2018). The limited data on female movement suggest that they also frequently disperse. Grasshopper sparrows disperse between breeding seasons as well; the percent of territorial males that return in any subsequent year at the Konza Prairie is 26% (Silber et al. 2023) and the percent of individuals resighted from the previous year ranges from 13.7% to 22.5%, which is at the low end of the 0%–57% return rates reported from throughout North America (Gill et al. 2006; Jones et al. 2007).

### 2.2 Field Methods

We estimated demographic rates for our IPM by mark‐recapture of adults and fledglings, as well as by following nests at KPBS and RP. Throughout each breeding season of 2013–2021, we captured adult (typically male) grasshopper sparrows in mist nets baited with a speaker playing a territorial male's song and marked them with a unique combination of one numbered, aluminum United States Geological Survey and three colored leg bands. Additionally, we captured females by placing a mist net directly in front of nests and flushing the bird into the net. We confirmed sex based on the presence of a cloacal protuberance (male) or a brood patch (female). We classified individuals as juveniles or adults based on plumage; we define juveniles as birds born in the year of capture, while adults were born in any year prior to capture. Because juveniles do not have cloacal protuberances or brood patches, the sex of juveniles was unknown unless they were molecularly sexed for other projects. We placed only a single metal numbered band on the leg of juveniles, but when they returned in subsequent years as adults, we recaptured them and added colored leg bands. Capture success for unmarked adult males was 100%. Once we found nests, capture success of females was also very high (~90%), but we occasionally aborted efforts to minimize disturbance at nests.

Field crews surveyed 18 pastures totaling 1643.8 ha every 10 days from mid‐April through late August so that each pasture was surveyed 8–10 times per breeding season. The pastures surveyed ranged in size from 27.9–226.3 ha and included three ungrazed × annual burn, three ungrazed × 2‐year burn, two bison grazed × annual burn, two bison grazed × 2‐year burn, two cattle grazed × annual burn pastures, and six cattle grazed × 3‐year patch burn pastures. Each survey was conducted by one or two observers, and routes varied between each survey to ensure all areas within each pasture were surveyed multiple times throughout the season. Using binoculars, scopes, and cameras, observers sighted and recorded the identity of individual birds. Sighting of unmarked birds triggered subsequent capture efforts. We did not include management regime in analyses because birds frequently moved between treatments; they were predominantly (> 58% of territories) found in patch‐burn‐grazed pastures, and because we were interested in the net effects of weather on birds independent of management.

We searched for nests throughout the breeding season in areas where males were defending territories. We located nests by observing adults carrying food and by flushing females off nests, either incidentally or by dragging a weighted rope over the top of the vegetation (Higgins et al. 1969). We visited nests every 2–3 days and recorded the number of grasshopper sparrow and brown‐headed cowbird ( *Molothrus ater* , a common brood parasite in NE Kansas) eggs or nestlings present until all nestlings successfully fledged or died. We marked nestlings at approximately Day 4 post hatch with a single, numbered metal band to enable identification post‐fledge. We were not always able to identify the nesting adult female (because they are secretive and can evade both capture and resight by running through the tall grasses), but we noted the total number of nesting females (both identified and not identified) for use in subsequent analyses (i.e., number of juveniles produced per nesting female). Our assumption that unidentified or unmarked females were separate individuals is unlikely to influence estimates because we found so few subsequent nests of our marked females; we found two or more nests for only 31 of the 277 marked females, and sometimes, those spanned more than 1 year.

### 2.3 Integrated Population Model

We collated the mark‐recapture and nest surveys into three data sets: annual population counts, a capture‐recapture/resighting matrix, and annual measures of productivity. Annual population counts consisted of the sum of unique individuals (adults and juveniles) observed at least once in the breeding season. We included all observed individuals in the capture‐recapture/resighting matrix except those with unknown IDs or adults of unknown sex. Juveniles of unknown sex were randomly assigned a sex based on a 1:1 offspring sex ratio common in passerines (Gowaty 1993). The productivity data set included the total count of females observed and the number of grasshopper sparrow juveniles in each year.

To estimate annual vital rates and population growth rates from these three datasets, we fit an integrated population model (IPM; Schaub et al. 2007). In an IPM, a state‐space model describes the population count data (Schaub and Kéry 2021). The state process represented a population projection model that considered two sexes and three stages (recruits, surviving adults, and immigrants). Recruits were individuals known to have been born in the study area in the year prior that returned to their natal site to breed. Surviving adults were > 1 year old who were known to have bred in the study area in any previous year, while immigrants were individuals that had never been banded at the study area in previous years. While we were unable to mark all individuals at the site each year, we did attempt to mark as many as possible, and assigning unmarked birds as immigrants is consistent with isotopic (W.A.B., unpublished data) and analytical (Silber et al. 2023) estimates of the rates of immigration in this system. We estimated stage‐specific abundance using binomial and Poisson distributions to account for demographic stochasticity (Schaub and Kéry 2021).

We used the capture‐recapture data to estimate apparent survival of both sexes and two age classes (juvenile and adult) with a Cormack‐Jolly‐Seber model (Schaub and Kéry 2021). We estimated productivity (i.e., number of fledglings produced by one adult female per year) using a regression model with a Poisson distribution fitted to the number of juveniles and females each breeding season (Schaub and Kéry 2021). We included a parameter as a denominator for fecundity in the regression model to account for known underestimation of the number of offspring produced each year. We included this parameter because, when studying post‐fledge survival in 2021 and 2022, only 0.04% of juveniles caught at the end of the breeding season were recaptures (i.e., birds banded as nestlings). This underestimation was a result of several methodological and biological factors, including the area of land surveyed relative to the number of observers, the difficulty of finding ground nests in grasslands, and the rapid pace of embryonic and nestling development in this species. A uniform prior was specified for the parameter ranging from 1 to 3, which accounts for observing between 33% and 100% of all juveniles due to not finding their nest each year.

We used results from other studies on grasshopper sparrows and knowledge of the local system to inform priors. We specified a uniform prior from 0.3 to 0.9 for the adult male and female apparent survival parameters based on a published range of adult male survival estimates (0.4–0.9; Bernath‐Plaisted et al. 2021). We applied a uniform prior from 0.1 to 0.5 for juvenile male and female apparent survival (i.e., young birds that survived and returned to the site the following year) because juvenile survival is estimated to be approximately half that of adults (Seigel 2009). The prior for adult male recapture probability was set as uniform from 0 to 1, reflecting the range of published return rates of 0% (Kaspari and O'Leary 1988) to 84% (Delany et al. 1995). Female and juvenile recapture rates were expected to be less than adult males (Small et al. 2009), so the prior for juvenile male recapture probability was specified as uniform from 0 to 0.5, while the priors applied to juvenile and adult female recapture probabilities were uniform from 0 to 0.3. The number of immigrants into a population is likely site specific and immigration rates have not been well characterized in grasshopper sparrows, so we set the limits of the immigration prior to be broad. Both priors for male and female immigration were uniform from 0 to 175 individuals, which was selected because the upper limit represents 50% of our initial population size estimates and reflects the proportion unmarked at the beginning of the study. Because grasshopper sparrows can have as many as 4.2 fledglings per nest (Davis et al. 2016) and have multiple nest attempts per year (e.g., can have an average of 3.5–4.8 nest attempts per year in West Virginia; Wray et al. 1982), we specified the prior for fecundity to be uniform from 0 to 14 offspring. We estimated the initial population size to be between 80 and 350 individuals. These estimates are ~2× the lowest and highest number of individuals counted within the previous five years from line transects conducted throughout the field site prior to 2013 in an independent study (Boyle 2023a, 2023b). We doubled the counts because almost all individuals counted were territorial males. We used Markov chain Monte Carlo (MCMC) to fit the model to the data, using three independent chains for 1,500,000 iterations (burn in = 750,000 and thin = 100). We fit the IPM and performed MCMC‐based diagnostics using the package *jagsUI* (Kellner 2021) in R version 4.1.1. This fitting resulted in estimates of annual demographic rates (adult and juvenile survival and immigration of males and females, fecundity) and annual estimated population sizes for each year t, $N_{t}$.

### 2.4 Sensitivity Analyses

Using the annual vital rates derived from the IPM, we constructed a simple stage‐based population model. We estimated population size (*N*) in time *t* as

$$N_{t}=I_{t}+\left(N_{t-1}\times \phi_{a,t}\right)+\left(N_{t-1}\times F_{t-1}\right)\times \phi_{j,t},$$

Using estimates of annual juvenile apparent survival (*ϕ*_*j,t*_) from *t*‐1 to *t*, adult apparent survival (*ϕ*_*a,t*_) from *t*‐1 to *t*, fecundity in the last breeding season (*F*_*t*‐1_) and number of immigrants observed at time *t* (*I*_*t*_). We calculated the annual population growth as $\lambda_{t}=\frac{N_{t}}{N_{t-1}}$. We then calculated the geometric mean of $\lambda_{t}$ across the seven annual transitions (i.e., $\lambda_{1}\lambda_{2}\ldots {\lambda_{7}}^{1/7}$), to estimate $\lambda_{s}$, population growth rate over the entire study period (Morris and Doak 2002).

We then used a perturbation approach (Morris and Doak 2002) to estimate sensitivity and elasticity of $\lambda_{s}$ to each of the vital rates (sex‐specific juvenile and adult apparent survival, male and female immigration, and fecundity). We did both because sensitivity analyses report the absolute contribution of each vital rate to population change, while elasticity analyses indicate the proportional contribution of each vital rate to population change. Elasticities are sensitivity rates that are scaled for each vital rate so multiple vital rates can be compared even if they do not have the same units (e.g., survival is a probability and fecundity is a number). We perturbed the annual estimates of each vital rate by 5%, one vital rate at a time, and then recalculated $\lambda_{s}$ (averaging the percent change in vital rates across years as in Louthan et al. 2022). To estimate elasticity, we calculated the proportional change in $\lambda_{s}$ for proportional change in each vital rate in a similar manner.

### 2.5 Weather Metrics

To assess the effect of weather on population growth (mediated through weather effects on vital rates), we calculated three weather metrics previously shown to be associated with grasshopper sparrow physiology (Freeman et al. 2023) and demography (Silber et al. 2023) that encompass local measures of temperature, humidity, and rainfall, as well as a regional, integrative measure of weather patterns based on rainfall anomalies related to the El Niño cycles. First, we extracted local high resolution (~4 km) Gridded Surface Meteorological (gridMET) data for our study site (Abatzoglou 2013). We extracted the maximum daily temperature (°C) and maximum daily relative humidity (%) for May 1–August 31 and calculated the average daily high wet bulb temperature (°C) for each breeding season (Stull 2011). Wet bulb temperature incorporates both air temperature and humidity, is always lower than the measured dry bulb temperatures, and is biologically relevant because the thermoregulatory costs of high air temperature increase with high humidity (Gerson et al. 2014). Second, because storms can affect survival, immigration and emigration, and productivity (Wingfield et al. 2017), we calculated the number of storms within each breeding season. We define a storm using metrics from Freeman et al. (2023); briefly, we classified a storm as a gridMET‐derived rainfall event that exceeded one standard deviation above the mean amount of rain (mean = 18.21 mm). Lastly, we included a broad, regional metric of breeding season temperature and precipitation: the El Niño‐Southern Oscillation Precipitation Index (ESPI). In Kansas, positive values indicate a wet and cold El Niño year while negative values indicate dry and warm La Niña summer (Curtis and Adler 2000). We summed May–August monthly ESPI data (from the University of Maryland Global Precipitation Climatology Project (2020)) for each breeding season and used this value (hereafter, “ESPI”), lagged 2 years, as a potential predictor for demographic rates. We included ESPI in this study in addition to the locally measured weather variables because (a) this climatic index captures covariation of multiple correlated weather variables at once, (b) it reflects weather over a broader region that likely influences dispersal in and out of the site, and (c) because in a previous study conducted in the same system, two‐year lagged ESPI correlated with emigration rates (Silber et al. 2023). We used these weather metrics (average daily high wet bulb temperature, number of storms, and ESPI) as predictor variables for demographic rates.

### 2.6 Path Analysis

We quantified the effects of weather metrics on demographic rates and of demographic rates on $\lambda_{t}$ using a path analysis, following the approach developed by Woodworth et al. (2017). We modeled all hypothesized linkages among the scaled weather metrics, the scaled sex‐ and age‐ specific vital rates (see Appendix S1 for hypothesized linkages), as well as between the vital rates and scaled $\lambda_{t}$ using multiple linear regressions. In total, the path analysis was composed of seven linear regressions: one of each scaled vital rate against each scaled annual weather metric, and one of scaled $\lambda_{t}$ against scaled annual vital rates. We fitted each model to each sample of the posterior distribution generated by the IPM which allowed for the uncertainty within the IPM to be carried forward (Woodworth et al. 2017). We ran each model within the path for 225,000 iterations, once for each value of the posterior distributions generated by the IPM, after which we calculated the mean estimate and the 95% credible intervals. We estimated the net effects of weather on $\lambda_{t}$ by calculating the product of the direct effects of each weather metric on a vital rate and that vital rate on population growth, summed across demographic rates.

### 2.7 Population Projections

The path analysis revealed that ESPI was the primary weather metric affecting population growth; ESPI had a 5× greater effect on $\lambda_{t}$ than the next most important weather metric. Thus, we wanted to use the relationship between historical ESPI and $\lambda_{t}$ to project (future) population growth rates as a function of (future) projected ESPI. However, GCMs do not project ESPI (because it is a record of ongoing weather). Thus, we needed to find a proxy for EPSI that was available for both historical conditions and for future projected conditions, so that wepath could use historical relationships to predict future dynamics. We used precipitation (lagged 2 years, as EPSI in the path analysis) as a proxy, derived from the historical gridMET data. The historical gridMET lagged precipitation data were moderately correlated with the historical lagged ESPI index (Pearson's correlation: *r* = 0.53), indicating that precipitation is a reasonable proxy for ESPI at our site. Importantly, the Multivariate Adaptive Constructed Analogs climate data (MACA; Abatzoglou and Brown 2012) are constructed in the same way as the gridMET data, allowing us to use historical relationships between $\lambda_{t}$ and weather to predict population growth rate as a function of projected weather. Thus, to estimate the relationship between historical ESPI and $\lambda_{t}$, we fit a linear regression to predict changes in $\lambda_{t}$ as a function of lagged precipitation.

We then used this regression to project population sizes under realistic future climate conditions. Namely, we predicted annual population growth rates from 2021 to 2100 using downscaled projections from the Multivariate Adaptive Constructed Analogs (MACA; Abatzoglou and Brown 2012), which provide downscaled site‐specific predicted climatic variables from suites of global circulation models (GCMs). Using coordinates from the middle of our study site, we constructed projections for all 18 GCMs available. Note that using a regression between lagged precipitation and annual population growth rate (rather than regressions between lagged precipitation and vital rates) does not allow for compensatory dynamics among vital rates (Oldfather et al. 2021). We found minimal evidence for opposing effects of weather variables on vital rates, meaning such compensatory dynamics are unlikely.

## 3 Results

### 3.1 Integrated Population Model

**FIGURE 1:**
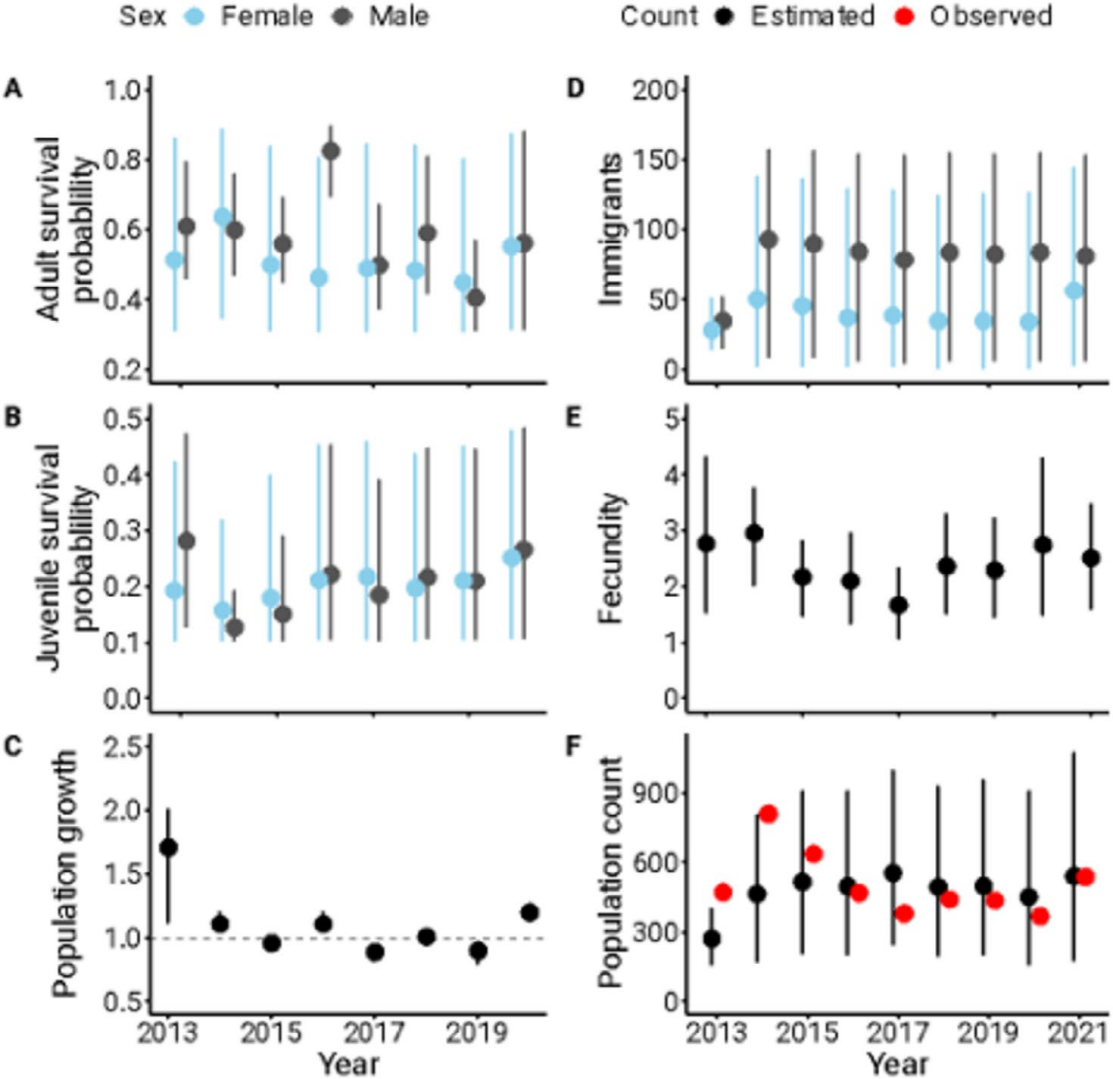
Annual estimates of vital rates and population counts of grasshopper sparrows ( *Ammodramus savannarum* ) including survival probabilities of adults (A) and juveniles (B), annual population growth rates (C), the number of immigrants (D), fecundity (i.e., the number of juveniles produced per female, E), and population counts (F). Dots represent the mean estimate, and the error bars represent the 95% credible intervals.

From 2013 to 2021, we captured 2898 unique individuals comprising 1640 adult males, 277 adult females, 36 juvenile males, 20 juvenile females, and 925 juveniles of unknown sex. Across the 9 years, we recaptured or resighted 2445 individuals at least once for a total of 3641 recaptures/resights. For adults, resight/recaptures ranged from 179 in 2021 to 376 in 2015 (mean across each year = 264.6 adults), while for juveniles it ranged from 43 in 2021 to 367 in 2024 (mean across each year = 109.6 juveniles). The average annual probability of apparent survival varied by sex and age class (adult male = 58%, adult female = 51%, juvenile male = 21%, juvenile female = 20%, Figure 1A,B). Immigration into the study area was high and varied by sex, with an estimated average of 79 males and 40 females joining the population each year (Figure 1D). Overall, the estimated number of individuals in the population fluctuated from a high of 810 in 2014 to a low of 369 in 2020 (Figure 1F) with no clear directional change over time. The estimated annual population growth rate was relatively high in the first year of the study (population growth rate in 2013 = 1.71), after which it oscillated around 1 (range for remaining years = 0.89–1.20, Figure 1C).

We located and monitored 498 nests over the 9 years of the study, 206 of which had known female parents (mean nests found per year = 62.3, range = 17 in 2020 to 117 in 2014). Females attempted 1–3 known clutches per year at the site, and nests contained an average of 3.32 grasshopper sparrow eggs (range = 0–7) with an average of 0.85 grasshopper sparrow nestlings successfully fledging per nest (range = 0–6). The total clutch size (grasshopper sparrow and brown‐headed cowbird eggs combined) ranged from 0–10 eggs and the total brood size (grasshopper sparrow and brown‐headed cowbird nestlings combined) ranged from 0–6 nestlings. The number of grasshopper sparrow juveniles known to be produced each breeding season ranged from 17 to 120 and the average number of juveniles produced per female per breeding season (i.e., fecundity) was estimated at 2.40 (Figure 1E).

### 3.2 Sensitivity Analyses

**FIGURE 2:**
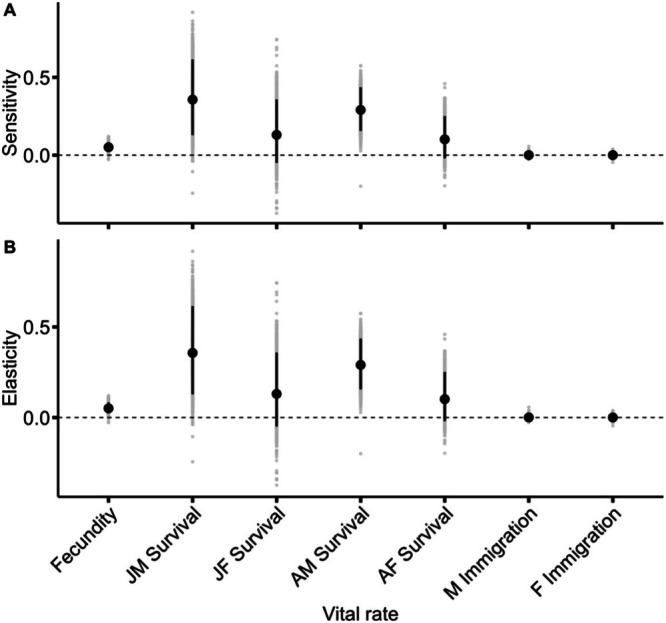
Percent change in the population growth rate (*λ*) of grasshopper sparrows ( *Ammodramus savannarum* ) given a 5% change in each vital rate each year (i.e., sensitivity; A). The proportional contribution, or percent change scaled for vital rate units, in the population growth rate (B). The population growth rate changes more in response to adult survival than in response to changes in juvenile survival, fecundity, or immigration. AF, adult female; AM, adult male; F, female; JF, juvenile female; JM, juvenile male; M, male.

Stochastic population growth rate, $\lambda_{s}$ was most sensitive to changes in adult male apparent survival (Figure 2). Sensitivity was highest for juvenile (0.36) and adult (0.29) male apparent survival, moderate for juvenile (0.13) and adult (0.10) female apparent survival, and lowest for fecundity (0.05) and immigration (< 0.01). The ranking of the elasticity values (i.e., proportional change in $\lambda_{s}$) were extremely similar to the sensitivity values (Figure 2).

### 3.3 Weather Summaries

Weather metrics were highly variable among the 9 years of study (Appendix S1). During the breeding season, average daily highs for wet bulb temperature stayed consistently around 27°C (range = 26.1°C–28.8°C). Total rainfall across the breeding seasons was highly variable, ranging from 347 mm in 2017 to 847 mm in 2019. Grasshopper sparrows were exposed to 4 (2018) to 16 (2019) storms (i.e., rainfall events exceeding 18.2 mm) between May and August each breeding season (mean number of storms per year = 9.2). ESPI was lowest in 2015 (−1.10) and highest in 2017 (2.68), which when lagged by 2 years, matched the extremes of the total rainfall.

### 3.4 Path Analysis

**FIGURE 3:**
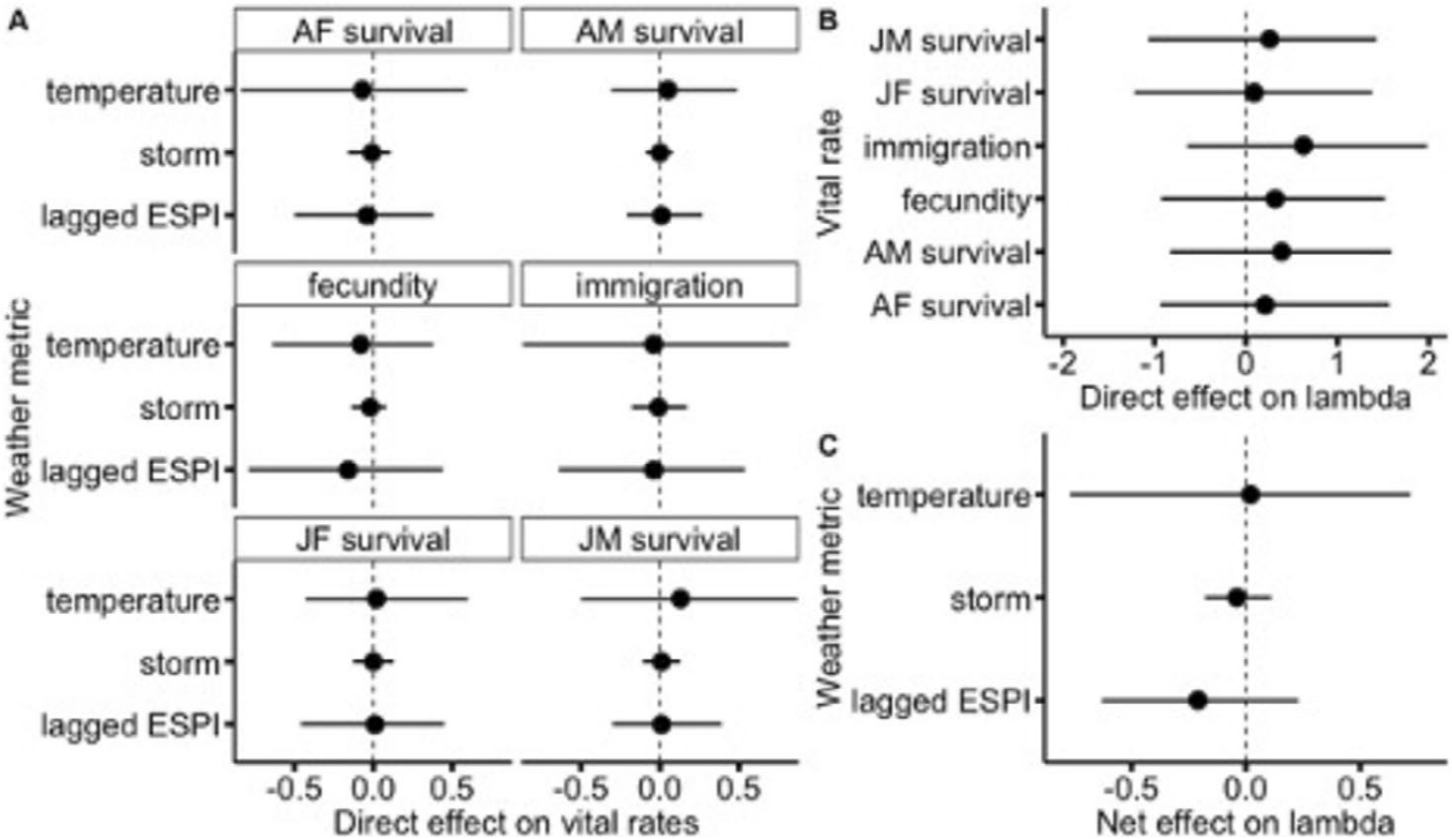
Estimates of the direct (A, B) and net (C) effects of weather and vital rates on the annual population growth rate of a population of grasshopper sparrows ( *Ammodramus savannarum* ) at Konza Prairie Biological Station, KS, USA. AF, adult female, AM, adult male; ESPI, El Niño‐Southern Oscillation Precipitation Index; JF, juvenile female; JM, juvenile male. Dots represent the mean estimate and the error bars represent the 95% credible intervals.

The path analysis indicated that immigration had the largest direct effect on $\lambda_{t}$ (mean estimate = 0.63, 95% credible interval (CI) = −0.64, 1.98) followed by adult male apparent survival (mean = 0.39, 95% CI = −0.83, 1.59). All direct relationships including effects of weather on vital rates and the effect of vital rates on $\lambda_{t}$ had credible intervals that substantially overlapped zero (Figure 3A,B; Appendix S1).

We estimated the net effect of each weather metric (average daily high wet bulb temperature, number of storms, and lagged ESPI) on annual population growth rate by multiplying the direct effect of the weather metric on each vital rate by the direct effect of that vital rate on $\lambda_{t}$, and then summing across vital rates, for each weather metric. Lagged ESPI had the strongest effect on $\lambda_{t}$ (compared to wet bulb temperature and number of storms; mean estimate = −0.21, 95% CI = −0.63, 0.23, Figure 3C; Appendix S1) where $\lambda_{t}$ was highest 2 years following a hot and dry year.

### 3.5 Population Projections

**FIGURE 4:**
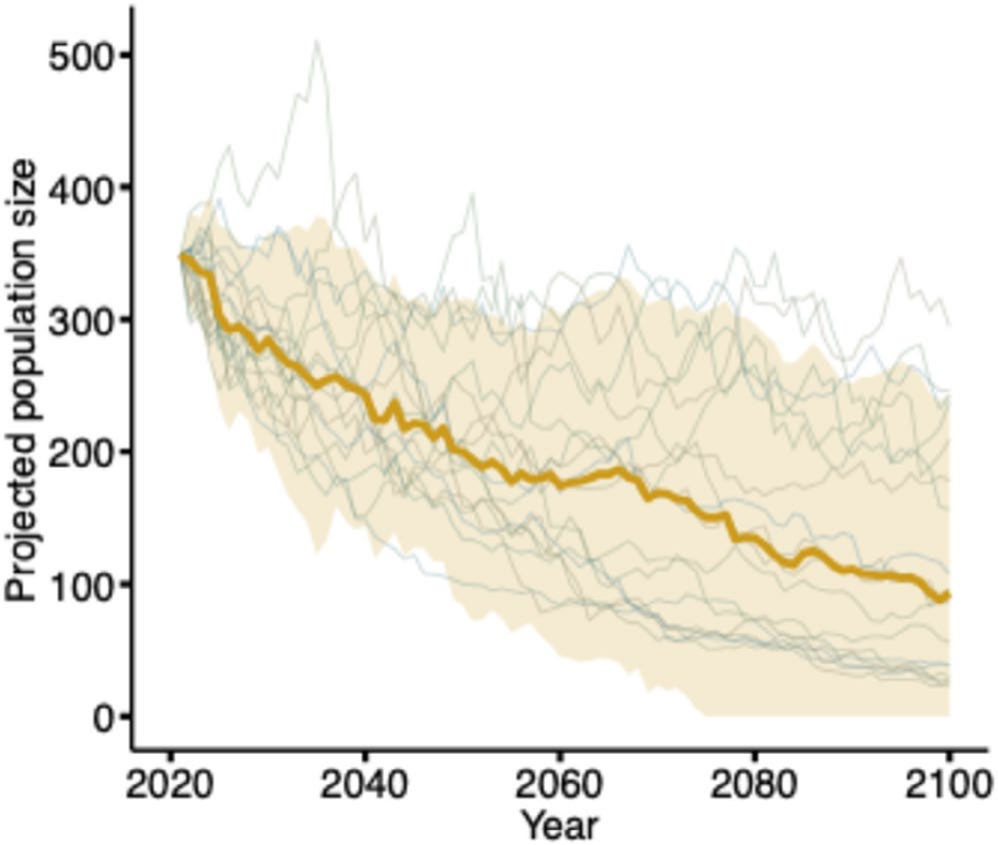
Projected population size of a population of grasshopper sparrows ( *Ammodramus savannarum* ) at Konza Prairie Biological Station, KS, USA (2021–2100). Future population sizes were calculated using projected breeding season precipitation lagged 2 years, derived from global climate models (GCMs). Each gray line represents population projections derived from a different GCM's climate projections. The yellow line and shaded region represent mean population size and 95% prediction interval for projected population size, respectively.

We projected annual population growth rate and size through 2100 using projected precipitation lagged by 2 years (a proxy for lagged ESPI) from 18 GCMs. The (arithmetic) mean annual population growth rate over the 2021–2100 period was projected to be 0.98 (95% CI = 0.86, 1.11). The projected population size declined under all climate scenarios (Figure 4). Annual population size averaged across the 18 GCM's projections (using the median) declined from the 2013 estimate of 539 individuals to 87 individuals by 2100, with two GCMs predicting the population size dropping below 100 individuals as early as 2055.

While the results shown in Figure 3 show perhaps only a weak relationship between the weather variables and vital rates, the population projection in Figure 4 shows a much more pronounced decline. On the surface, there may appear to be a discrepancy between the path analysis (e.g., results shown in Figure 3) and the population projection (Figure 4). The idea that one can look at the credible intervals and determine if a variable like weather has an impact because the credible intervals overlap zero (e.g., as in Figure 3) may miss some key features of the marginal posterior distribution (e.g., skewness) and certainly misses features of the joint posterior distribution (e.g., correlation among parameters). The population projection shown in Figure 4 is another way to visualize a transformation of the joint posterior in a way that may provide different insights.

## 4 Discussion

Our study species' population growth rate is predicted to decrease in a future climate. While credible intervals for hypothesized relationships between aspects of weather and demographic rates were broad and overlapped zero, population size appears to be related to precipitation, and the net declines predicted are likely due to high interannual variation in predicted precipitation. The annual population growth rates observed during our study were highly variable and exceeded the common bounds of long‐term variation in population growth rates in other wildlife (annual population growth rates in our population: 0.89–1.71, common bounds: 0.95–1.05, Koons et al. 2006). Given that we see no evidence for a convex relationship between precipitation and population growth rate (Appendix S1, Drake 2005), this variability should decrease stochastic population growth rate (Morris and Doak 2002). Consistent with this expectation, we see declines in population sizes under future climate conditions. These results predict that grasshopper sparrows may be locally extirpated within the next century even without considering the well‐known additional threats of habitat loss, degradation, and fragmentation (Herkert 1998; Stanton et al. 2018; Rosenberg et al. 2019). Our results are consistent with analyses of density and reproductive metrics that predict grasshopper sparrows are not regionally viable (With et al. 2008).

Interannual variation in the annual population growth rate was most sensitive to changes in adult apparent survival (i.e., the product of true survival and movement), which in this population is shaped more by emigration than true survival (Silber et al. 2023). Thus, high variation in local population growth rate is a product of movement in and out of the area (Silber et al. 2023), with previous work suggesting that return rates are highest following years with high values of ESPI (Silber et al. 2024). Furthermore, the causal links between lagged ESPI and grasshopper sparrow demography appear to be mediated by vegetation structure. Namely, grasshopper sparrows select quite specific structural attributes for breeding territories, and those attributes are also related to lagged rainfall (Silber et al. 2023, 2024). In addition, nest success in grasshopper sparrows is associated with vegetation structure mediated, in part, by precipitation in previous years (Ruth and Skagen 2018), with juveniles moving to areas with higher vegetation cover (Small et al. 2015; Guido 2020). Finally, vegetation structure is associated with multiple vital rates in this species and their communities (e.g., Anderson et al. 2015; Giovanni et al. 2015). Overall, in this study, lagged ESPI had a 5–10 times larger net effect on the population growth rate than the other weather variables we included. These studies highlight the need for large‐scale, regional analyses to determine why and where birds are moving to elucidate the mechanisms underlying meta‐population dynamics.

Our study has at least two limitations. First, although long from the perspective of most intensive field studies of wild animal populations, the 9 years of this study represent a relatively short demographic study, which might prevent us from accurately estimating variability in precipitation and in precipitation effects on vital rates. Because the habitat, weather, and population are highly dynamic in this system, 9 years may not provide enough data to explore fine‐scale relationships between precipitation and individual performance. In particular, 9 years may be insufficient to characterize the concavity of the precipitation‐population growth rate relationship, which is critical to predicting effects of precipitation variability on population growth rate (Lawson et al. 2015). Second, due to the secretive nature of females and the difficulty of finding renests, considerable uncertainty remains around fecundity estimates. Nevertheless, this combination of an IPM, sensitivity and path analyses, and population projection is the most comprehensive study of the population dynamics of a grassland bird at a single site to date, and represents a significant advancement in the road to recovery of a declining species. Our incorporation of informative priors is also an important step forward when compared to other demographic studies (e.g., Woodworth et al. 2017) and draws on years of in‐depth knowledge to strengthen inference.

Developing IPMs is a critical step in guiding effective conservation efforts (Zipkin and Saunders 2018). Often, the focus of applied studies is on local habitat selection; although it is important to understand local conditions most strongly associated with occupancy, we need a broader perspective on species' relationships with their environment. Demographic models such as this one aid in understanding which vital rates are most important for persistence, which represents a key step in recovering North American birds (North American Bird Conservation Initiative 2022). Such work paves the way for the next step, which is to implement targeted conservation actions and a relatively new field of inquiry such as active climate change mitigation via managing for refugia. In this population, we recommend mitigating climatic drivers through careful and year‐specific habitat management in ways that will increase fecundity, promote immigration, and suppress emigration (Silber et al. 2024). We know from prior work that woody plant suppression (often achieved through burning) is crucial to the persistence of grassland obligate birds (Andersen and Steidl 2019). However, the majority of such species also depend on patches of unburned vegetation in which to nest, which underlies recommendations for management that create habitat patchiness (Bernath‐Plaisted et al. 2023). This study highlights actions that are likely to have positive demographic impacts for populations of grasshopper sparrows in tallgrass prairies; in the wettest years, we recommend elevated grazing intensities to mitigate the negative lagged effect of high rainfall, which is likely mediated by the effects of rain on the height and density of grassland vegetation in future years. Given the propensity for movement in grassland birds (Jones et al. 2007), and the ultimate effects on local population persistence, management plans and actions at regional scales are imperative. Pastures with heterogeneous vegetation and aggressive suppression of woody encroachment best support diverse communities of grassland‐dependent species (Wiens 1969; Bakker 2003). Furthermore, combining land management efforts on the breeding grounds with ongoing work at grasshopper sparrow overwintering sites (e.g., Macías‐Duarte et al. 2017; Pérez‐Ordoñez et al. 2022) will be an important step in the recovery of the population.

Despite the variability in demographic rates and responses to weather variables, the overall declines predicted support the idea that in climatically variable environments, animal populations are regulated by environmental conditions (Andrewartha and Birch 1954; Drake and Lodge 2004; Jenouvrier et al. 2015). By exploring the associations between grassland‐obligate birds and weather, our study provides nuanced insight into the plight of these imperiled birds and provides an example of how such insight paves the way for action that mitigates the future predicted for them under anticipated climate scenarios. However, the hopeful side of this story is that their population growth is likely mediated by vegetation responses. Thus, management can be effective in compensating for those climatic drivers in other ways to achieve the suite of conditions required for population growth.

## Author Contributions

**N. E. Freeman:** data curation (equal), formal analysis (equal), methodology (equal), visualization (equal), writing – original draft (lead), writing – review and editing (equal). **K. M. Silber:** data curation (equal), formal analysis (equal), investigation (equal), writing – original draft (supporting), writing – review and editing (equal). **A. M. Louthan:** formal analysis (equal), investigation (equal), supervision (equal), writing – original draft (supporting), writing – review and editing (equal). **T. J. Hefley:** conceptualization (equal), formal analysis (supporting), funding acquisition (equal), project administration (equal), supervision (equal), writing – review and editing (equal). **W. A. Boyle:** conceptualization (equal), data curation (equal), funding acquisition (equal), investigation (equal), project administration (equal), supervision (equal), writing – original draft (supporting), writing – review and editing (equal).

## Ethics Statement

We followed all guidelines for the care and use of animals as well as institutional, state, and federal regulations throughout our study (Kansas State University IACUC permits #3260, 3733, and 4250 and North American Bird Banding Laboratory permit #23836).

## Conflicts of Interest

The authors declare no conflicts of interest.

## Supporting information


**Data S1:** ece372195‐sup‐0001‐DataS1.zip.

**Appendix S1:** ece372195‐sup‐0002‐AppendixS1.zip.

## Data Availability

Analyses in this study can be replicated using the provided R code (supporting information file S1 Supinfo2.R) and the data (mark recapture data: Supinfo5.csv; fecundity data: Supinfo4.csv; population count data: Supinfo3.csv; weather data: Supinfo8.csv and Supinfo7.csv; global climate change data: Supinfo6.csv). The metadata for the datasets can be found in Supinfo1 document.
