# Supplementary material for: Climatic Variability Threatens Population Growth and Persistence of a Declining Grassland Songbird
Source: Ecol Evol. 2025 Nov 24;15(11):e72195. doi: 10.1002/ece3.72195 (PMC12643823; doi:10.1002/ece3.72195)
Supplement: Supplementary file 1 — Data S1: ece372195‐sup‐0001‐DataS1.zip. [file ECE3-15-e72195-s002.zip › ece372195-sup-0001-DataS1/ece372195-sup-0001-Supinfo1.docx]

**Climatic variability threatens population growth and persistence of a declining grassland songbird**

Using detailed demographic data collected between 2013-2021 from grasshopper sparrows (*Ammodramus savannarum*) at the Konza Prairie Biological Station, Kansas, USA, we developed an integrated population model to estimate survival, immigration, fecundity and population growth. Then, we conducted path and sensitivity analyses to assess the effect of variability in weather on vital rates and population growth and to understand how the vital rates were shaping population growth, respectively. Finally, we conducted projection analyses to determine how the population will fare under predicted climate scenarios.

Analysis was conducted in R Studio (R version 4.1.1). All packages used are listed in the provided code.

Files included are one R script containing code for the IPM, path, sensitivity and projection models and six data files in order of their use in the code:

1. **ece372195-sup-0002-Supinfo2.R** – this file includes the entire R script for the IPM, path analysis, sensitivity analysis and projection model
2. **ece372195-sup-0005-Supinfo5.csv** – this file is a matrix of presence and absence of all individuals in the study. Here is the metadata for the columns:
   1. ID = the bird’s unique ID, either as a color band combination (e.g. GK-SP) if they received color bands or their metal band number (e.g. 2443 12343) if they did not get marked with color bands
   2. 2013-2020 = a 0 means they were not present that breeding season while a 1 means they were observer that breeding season
   3. Capture.year = the year they were first captured and marked
   4. Sex = male or female
   5. Age = juvenile (born that breeding season) or adult (born in a previous breeding season)
3. **ece372195-sup-0004-Supinfo4.csv** – this file summarizes the number of offspring and breeding females by year. Here is the metadata for the columns:
   1. Year = the year of the count
   2. Fledglings = the number of fledglings that fledged that year
   3. Females = the number of females observed that year
4. **ece372195-sup-0003-Supinfo3.csv** – this file is a summary of the numbers of individuals observed each year of the study broken down by sex and age. Here is the metadata for the columns:
   1. Adult.F = the number of adult females observed in that year
   2. Adult.M = the number of adult males observed in that year
   3. Juvenile.F = the number of juvenile females observed in that year
   4. Juvenile.M = the number of juvenile males observed in that year
   5. Total = the total number of individuals observed in that year
   6. Year = the year of the count
5. **ece372195-sup-0008-Supinfo8.csv** – this file is a summary of the weather and climate by year (breeding season = May 1-Aug 31). Here is the metadata for the columns:
   1. Year = the year of the study
   2. SevereStorms = the number of severe storms during the breeding season (= when rainfall during a single rainfall event exceeded one standard deviation above the mean, which meant rainfall exceeding 18.21 mm). Source was the precipitation data on gridMET: https://www.climatologylab.org/datasets.html
   3. AveHighWBulb = the mean daily high wet bulb temperature for the breeding season. Source was the maximum temperature and maximum humidity data on gridMET: https://www.climatologylab.org/datasets.html
   4. TwoYearLagESPI = summed monthly El Niño-Southern Oscillation precipitation index (ESPI) values lagged by two years (e.g. the breeding season in 2013 is linked with the EPSI values from 2011). Source: University of Maryland Global Precipitation Climatology Project http://gpcp.umd.edu/espi.htm
6. **ece372195-sup-0007-Supinfo7.csv** – this file is the population growth rate over time and lagged precipitation data. This was used to understand the relationship between
   1. Year = the year of the study
   2. Lambda = the population growth rate from each year estimated by the IPM
   3. Breed_lag2 = sum of the breeding season (May 1-Aug 31) precipitation in mm lagged by two years (e.g. the breeding season in 2014 is linked with the precipitation values from 2012). Source was the precipitation data on gridMET: https://www.climatologylab.org/datasets.html
7. **ece372195-sup-0006-Supinfo6.csv** – this file contains estimated data from 18 global climate change models using a Multivariate Adaptive Constructed Analogs (MACA) approach. This was used in the projection models. Source: https://www.climatologylab.org/maca.html. Here is the metadata for the columns:
   1. Yyyy-mm-dd = the date from January 1^st^ 2019 to December 31^st^ 2099
   2. The remaining 18 columns are the data for each of the 18 climate models broken down by date
