# Supplementary material for: Climatic Variability Threatens Population Growth and Persistence of a Declining Grassland Songbird
Source: Ecol Evol. 2025 Nov 24;15(11):e72195. doi: 10.1002/ece3.72195 (PMC12643823; doi:10.1002/ece3.72195)
Supplement: Supplementary file 2 — Appendix S1: ece372195‐sup‐0002‐AppendixS1.zip. [file ECE3-15-e72195-s001.docx]

**Appendix A for *Climatic variability threatens population growth and persistence of a declining grassland songbird.***

Hypothesized relationships between weather metrics and vital rates (table A1) and the structure of the associated path analysis (figure A1).

**Tables**

**Table A1**. Hypothesized relationships between weather and vital rates of a population of grasshopper sparrows (*Ammodramus savannarum*) in northeastern Kansas, USA during the breeding season (May to August). The weather metrics considered include the number of storms, the average daily high wet bulb temperature (°C), and the summed monthly El Niño-Southern Oscillation Precipitation Index (ESPI) lagged by two years.

| **Weather metric** | **Proposed hypotheses** | **Predicted response** |
| --- | --- | --- |
| storms | storms are energetically costly and could impact survival | more storms lower survival |
|  | storms can lead to nest failure via flooding or abandonment | more storms lower fecundity |
|  | storms may impede movement of individuals | more storms reduce immigration |
| temperature | excessive heat could impact survival if at thermoregulatory limits | hotter temperatures lower survival |
|  | excessive heat is energetically costly and could impact provisioning and offspring survival | hotter temperatures lower fecundity |
|  | excessive heat is energetically costly, could impact resources, and could impact movement and choice to settle | hotter temperatures reduce immigration |
| lagged ESPI | bottom-up effects of weather two years prior on vegetation and prey availability could alter exposure to predators and impact whether individuals choose to return | prior wet conditions increase survival by increasing concealment and site fidelity |
|  | bottom-up effects of weather two years prior on vegetation and prey availability could impact nest placement and success | prior wet conditions increase fecundity by increasing nest concealment and prey availability |
|  | bottom-up effects of weather two years prior on vegetation and prey availability could impact whether individuals choose to settle | prior wet conditions increase immigration via vegetation structure and increased prey availability |

**Figures**


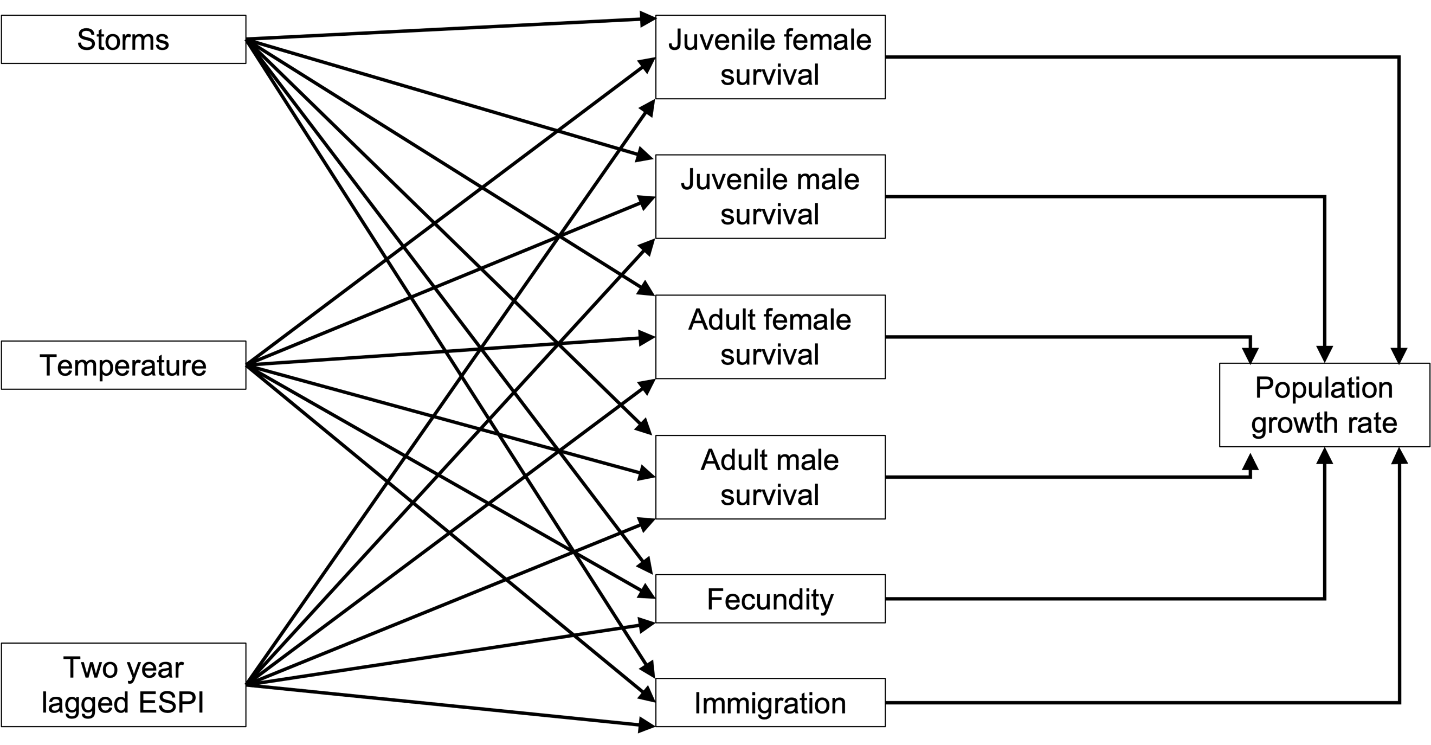


**Fig.A1.** Diagram of the path model fit to assess the direct and indirect relationships between annual weather metrics (the number of storms, the average daily high wet bulb temperature (°C), and the summed monthly El Niño-Southern Oscillation Precipitation Index (ESPI) lagged by two years), annual vital rates, and the annual population growth rate in a population of grasshopper sparrows (*Ammodramus savannarum*) in northeastern Kansas, USA during the breeding season (May to August).

**Appendix B for *Climatic variability threatens population growth and persistence of a declining grassland songbird.***

Change in temperature, precipitation, ESPI and number of storms throughout our study (Fig. B1).

**
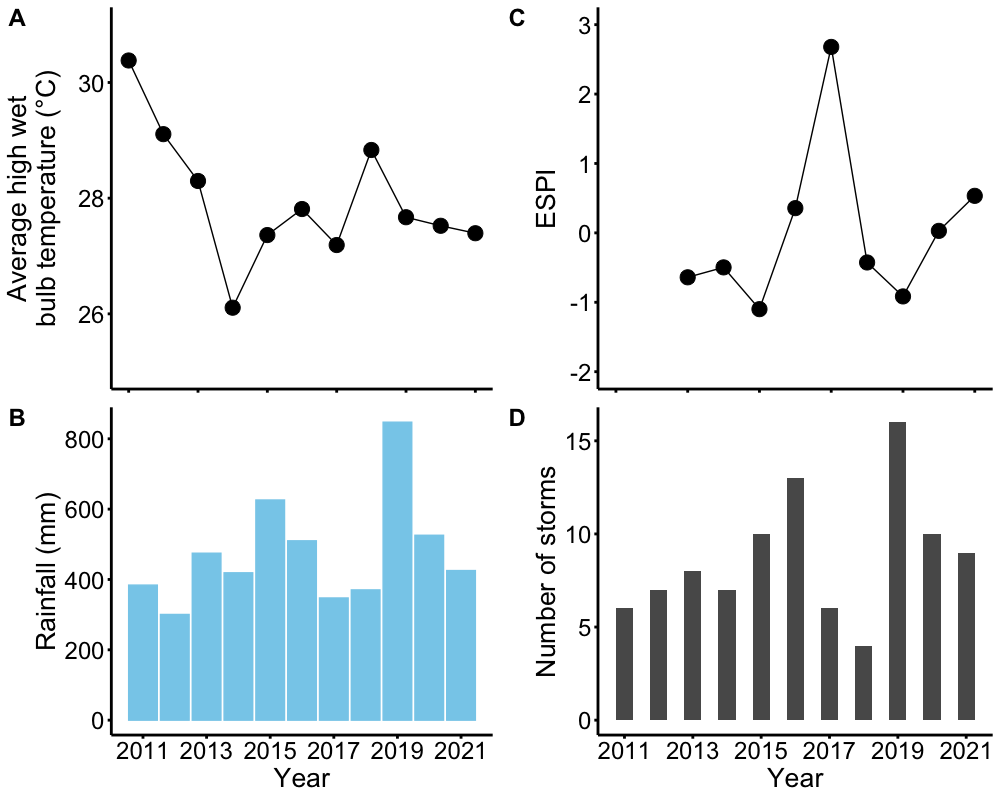
**

**Fig. B1.** Change in average daily high wet bulb temperature (°C, A), total precipitation (mm, B), the monthly summed El Niño-Southern Oscillation Precipitation Index (ESPI) lagged by two years (C), and the number of storms (D) across the breeding seasons (May to August) of 2011–2021 at Konza Prairie Biological Station, KS, USA.

**Appendix C for *Climatic variability threatens population growth and persistence of a declining grassland songbird.***

Summary of the direct (table C1) and indirect (table C2) relationships from the path analysis used to assess the relationships between weather, vital rates and population growth rate of grasshopper sparrows.

**Table C1**. Mean estimates and the lower and upper limits of the 95% credible interval from a path model fitted to assess the direct relationships between annual weather metrics (the number of storms, the average daily high wet bulb temperature (°C), and the summed monthly El Niño-Southern Oscillation Precipitation Index (ESPI) lagged by two years), annual vital rates, and annual population growth rates of a population of grasshopper sparrows (*Ammodramus savannarum*) in northeastern Kansas, USA during the breeding season (May to August).

| **Predictor** | **Response** | **Mean** | **Lower** | **Upper** |
| --- | --- | --- | --- | --- |
| storm | juvenile female survival | 0.00 | -0.13 | 0.13 |
|  | adult female survival | -0.01 | -0.16 | 0.11 |
|  | juvenile male survival | 0.01 | -0.11 | 0.13 |
|  | adult male survival | 0.00 | -0.09 | 0.08 |
|  | fecundity | -0.02 | -0.14 | 0.08 |
|  | immigration | -0.01 | -0.18 | 0.17 |
| temperature | juvenile female survival | 0.02 | -0.43 | 0.60 |
|  | adult female survival | -0.07 | -0.84 | 0.59 |
|  | juvenile male survival | 0.13 | -0.50 | 0.87 |
|  | adult male survival | 0.05 | -0.31 | 0.49 |
|  | fecundity | -0.08 | -0.64 | 0.38 |
|  | immigration | -0.04 | -0.87 | 0.82 |
| lagged ESPI | juvenile female survival | 0.01 | -0.46 | 0.45 |
|  | adult female survival | -0.04 | -0.50 | 0.38 |
|  | juvenile male survival | 0.01 | -0.30 | 0.39 |
|  | adult male survival | 0.01 | -0.21 | 0.27 |
|  | fecundity | -0.16 | -0.79 | 0.44 |
|  | immigration | -0.04 | -0.64 | 0.54 |
| juvenile female survival | population growth rate | 0.09 | -1.22 | 1.38 |
| adult female survival |  | 0.21 | -0.94 | 1.57 |
| juvenile male survival |  | 0.26 | -1.07 | 1.43 |
| adult male survival |  | 0.39 | -0.83 | 1.59 |
| fecundity |  | 0.32 | -0.93 | 1.52 |
| immigration |  | 0.63 | -0.64 | 1.98 |

**Table C2**. Mean estimates and the lower and upper limits of the 95% credible interval from a path model fitted to assess the indirect relationships between annual weather metrics (the number of storms, the average daily high wet bulb temperature (°C), and the summed monthly El Niño-Southern Oscillation Precipitation Index (ESPI) lagged by two years) and the annual population growth rate of a population of grasshopper sparrows (*Ammodramus savannarum*) in northeastern Kansas, USA during the breeding season (May to August).

| **Predictor** | **Response** | **Mean** | **Lower** | **Upper** |
| --- | --- | --- | --- | --- |
| storm | population growth rate | -0.04 | -0.18 | 0.11 |
| temperature |  | 0.02 | -0.77 | 0.72 |
| lagged ESPI |  | -0.21 | -0.63 | 0.23 |

**Appendix D for *Climatic variability threatens population growth and persistence of a declining grassland songbird.***

The relationship between population growth rate and precipitation lagged by two years (Fig. D1).


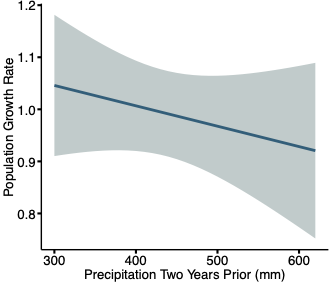


**Fig.D1.** Relationship between breeding season precipitation two years prior and the population growth rate of a population of grasshopper sparrows (*Ammodramus savannarum*) at Konza Prairie Biological Station, KS, USA (2014**–**2021). The population is expected to decrease following wetter conditions. The line represents the expected population growth rate and the shaded region represents the 95% credible intervals.
